# Circadian Rest and Activity Rhythms and Cognitive Change in the Baltimore Longitudinal Study of Aging

**DOI:** 10.1093/geroni/igab046.1722

**Published:** 2021-12-17

**Authors:** Yang An, Sarah Wanigatunga, Vadim Zipunnikov, Mark Wu, Eleanor Simonsick, Susan Resnick, Adam Spira, Jill Rabinowitz

**Affiliations:** 1 NIA, Baltimore, Maryland, United States; 2 Johns Hopkins, Baltimore, Maryland, United States; 3 Department of Biostatistics, Bloomberg School of Public Health, Johns Hopkins, Baltimore, Maryland, United States; 4 Johns Hopkins University School of Medicine, Baltimore, Maryland, United States; 5 National Instute on Aging/NIH, Baltimore, Maryland, United States; 6 National Institute on Aging, Baltimore, Maryland, United States; 7 Johns Hopkins Bloomberg School of Public Health, Baltimore, Maryland, United States

## Abstract

Alterations in 24-hour movement patterns, or circadian rest/activity rhythms (RARs), commonly occur with aging. Using linear mixed effects (LME) modeling, we examined associations of baseline RARs with longitudinal change in cognition. Participants (N=424; 47% male, baseline age 72.8±10.1 years) were from the Baltimore Longitudinal Study of Aging and completed 5.6±0.8 nights of wrist actigraphy at baseline. Tests of memory, executive function, attention, language, and visuospatial ability were administered at baseline and subsequent visits (3.7±1.7 years of follow-up in those with >1 visit (n=295)). In unadjusted random intercept and slope LME models, greater RAR stability predicted slower memory decline, and higher activity during participants’ least active 5 hours (L5) predicted slower decline in visuospatial ability. After covariate adjustment, higher activity in participants’ most active 10 hours (M10) and higher L5 predicted slower decline in visuospatial ability (p<.05). Further research is needed on RARs as risk factors for later-life cognitive decline.

